# Advances in Targeting Cutaneous Melanoma

**DOI:** 10.3390/cancers13092090

**Published:** 2021-04-26

**Authors:** Dimitri Kasakovski, Marina Skrygan, Thilo Gambichler, Laura Susok

**Affiliations:** 1 European Center for Angioscience (ECAS), Medical Faculty Mannheim, Heidelberg University, 68167 Mannheim, Germany; d.kasakovski@dkfz-heidelberg.de; 2 Division of Vascular Oncology and Metastasis, German Cancer Research Center Heidelberg (DKFZ-ZMBH Alliance), 69120 Heidelberg, Germany; 3 Department of Dermatology, Skin Cancer Center, Ruhr-University Bochum, 44801 Bochum, Germany; m.skrygan@klinikum-bochum.de (M.S.); t.gambichler@klinikum-bochum.de (T.G.)

**Keywords:** skin cancer, melanoma, cancer therapy, immunotherapy, targeted therapy, intratumoral therapy, combination therapy

## Abstract

**Simple Summary:**

Cutaneous Melanoma (CM), arising from pigment-producing melanocytes in the skin, is an aggressive cancer with high metastatic potential. While cutaneous melanoma represents only a fraction of all skin cancers (<5%), it accounts for most skin-cancer-related deaths worldwide. Immune checkpoint inhibition has been the first therapeutic approach to significantly benefit patient survival after treatment. Nevertheless, the immunosuppressive tumor microenvironment and the intrinsic and acquired treatment resistance of melanoma remain crucial challenges. Combining local and systemic treatment offers the potential to augment therapeutic response and overcome resistance, although, complex drug combinations can harbor an increased risk of immune-related adverse events. The aim of this review is to give current insight into studies combining systemic and local therapeutic approaches to overcome drug resistance, prime melanoma cells for therapy, and improve overall treatment response in CM patients.

**Abstract:**

To date, the skin remains the most common cancer site among Caucasians in the western world. The complex, layered structure of human skin harbors a heterogenous population of specialized cells. Each cell type residing in the skin potentially gives rise to a variety of cancers, including non-melanoma skin cancer, sarcoma, and cutaneous melanoma. Cutaneous melanoma is known to exacerbate and metastasize if not detected at an early stage, with mutant melanomas tending to acquire treatment resistance over time. The intricacy of melanoma thus necessitates diverse and patient-centered targeted treatment options. In addition to classical treatment through surgical intervention and radio- or chemotherapy, several systemic and intratumoral immunomodulators, pharmacological agents (e.g., targeted therapies), and oncolytic viruses are trialed or have been recently approved. Moreover, utilizing combinations of immune checkpoint blockade with targeted, oncolytic, or anti-angiogenic approaches for patients with advanced disease progression are promising approaches currently under pre-clinical and clinical investigation. In this review, we summarize the current ‘state-of-the-art’ as well as discuss emerging agents and regimens in cutaneous melanoma treatment.

## 1. Background

Cancer of the skin is considered a growing epidemic among Caucasians in the western world, with an alarming increase of non-melanoma skin cancer and cutaneous melanoma (CM) incidence rates of up to 44% over the last decade [1,2,3]. Moreover, increased accumulated exposure to ultraviolet (UV) radiation of the sun over lifespan due to growing life expectancy and the global climate crisis are considered major catalysts of increased incidence and mortality of skin cancer. Whilst CM is still most common in the male population aged 50–70 years, rates of CM in young adults, specifically in young women, are constantly increasing over the last years. Although recently approved novel targeted and immunotherapeutic approaches for CM treatment have been able to contain mortality rates of melanoma patients, it is predicted that nearly half a million people will be diagnosed with CM by 2040 with an increase in incidence of 62% and an increase of mortality of up to 74% [4,5,6]. In contrast to non-melanoma skin cancer (0.69) the age-standardized annual mortality rate for CM is about 1.5 (per 100,000 population) in the United States. These alarming numbers make the development of novel targeted therapeutic options and constant adjustments of the current state-of-the-art regimens essential in successful CM treatment. Several combinations involving immune checkpoint inhibition (ICI), targeted therapy through mutant-BRAF inhibition, intratumoral application of immunomodulators, oncolytic viruses, and anti-angiogenic approaches are being trialed to prime the anti-tumor response, enhance the sensitivity of CM cells to therapeutic interventions, and overcome therapy resistance of mutant cancer cells. 

**Figure 1:**
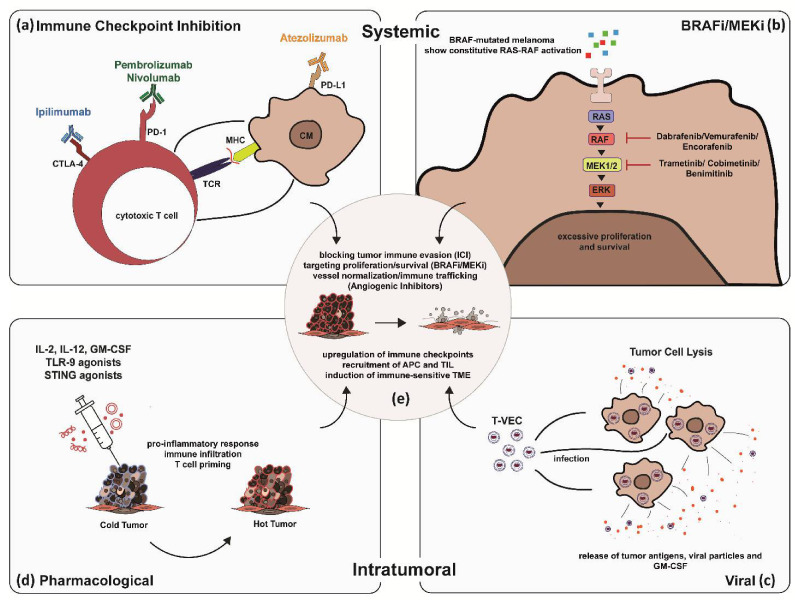
Synergistic effects of combined systemic and intratumoral therapy in skin cancer treatment exemplified by CM. Systemic immune checkpoint inhibition (**a**–**e**): Blocking of immune checkpoints on cytotoxic T Lymphocytes (CTL) and CM cells disrupting tumor immune evasion (**a**). Systemic BRAFi/MEKi: Blocking overactivation of RAS-RAF signaling pathway in BRAF-mutated melanoma by BRAF and MEK inhibitors (**b**). Intratumoral application of genetically engineered oncolytic viral particles to express checkpoint inhibitors on CM cells, force production and release of tumor antigens and GM-CSF to prime T cell immune response and induce tumor cell lysis (**c**). Intratumoral pharmacological application of IL-2, IL12, GM-CSF, TLR-9 and STING agonists: Priming locally advanced CM to become immunogenic (**d**).

Here, we describe current intervention strategies in CM and give pre-clinical insight into research avenues of future combination therapies in CM (Figure 1a–e).

## 2. Immune Checkpoint Inhibition

Understanding the process of immune-surveillance, or more specifically the capability of the immune system to identify and target neoplastic cells for destruction, revolutionized the treatment of a range of solid tumors [7,8]. Tumor cells are capable of hijacking the patients’ T cell immune checkpoints, such as cytotoxic T-lymphocyte associated protein 4 (CTLA-4) and programmed cell death protein 1 (PD-1), which act as negative regulators of immune response by surface expression of members of the B7 family of immune-regulatory ligands and B7 homologs such as programmed cell death ligand 1 (PD-L1) and programmed cell death ligand 1 (PD-L2) [9,10,11] (Figure 1a). Tumor cells thus use a mechanism, which inherently is meant to protect the immune system from aberrant auto-immune responses. T cell receptor (TCR)-dependent T cell activation is mediated by several co-stimulatory signals, most notably the CD28 surface receptor, which is highly expressed at basal levels. By contrast, CTLA-4 is only induced following antigen presentation and directly opposes CD28 stimulation [12,13,14]. Similarly, PD1 is only induced after TCR stimulation, and whilst CTLA-4 mostly acts within lymphoid organs, PD1 functions predominantly within peripheral tissues [12,15]. Other immune checkpoints include T cell immunoglobulin mucin-3 (TIM-3), which regulates T cell tolerance by inhibiting expansion and promoting apoptosis of Th1 and Th17 cells, thus leading to CD8+ T cell depletion. Furthermore, lymphocyte activated gene-3 (LAG-3), T cell immunoglobulin, and immunoreceptor tyrosine-based inhibitory motif (TIGIT), Glucocorticoid-induced TNFR family-related gene (GITR) and V-domain Ig suppressor of T cell activation (VISTA) contribute to activation and inhibitory function of regulatory T cells [16,17,18]. Whilst targeted therapy is generally associated with high but short-term treatment response rates in skin cancer patients, immune checkpoint blockade has mostly shown lower but more durable responses [19,20,21].

Remarkably, recent treatment of skin cancer with immune checkpoint inhibitors (ICI) was particularly successful with high and long-term overall response rates (ORR) (40–60%). Approved and currently in-use ICI for skin cancer treatment include cytokines that target the IL-2 and IFNAR1/2 pathways such as aldesleukin and interferon/peginterferon alfa-2b, PD-1 inhibitors such as pembrolizumab and nivolumab, PD-L1 inhibitors such as atezolizumab, and the first clinically approved monoclonal antibody for CM, ipilimumab, that targets CTLA-4 [22]. Ipilumab, although having a relatively low ORR of <20%, was the first therapeutic option to improve overall survival in metastatic melanoma patients significantly and is thus predominantly used in melanoma treatment [23,24]. Treatment of melanoma was the first to gravely benefit from the approval of immune checkpoint blockade, with ICI being the first therapy to significantly improve overall survival (OS) in patients with advanced disease states. The early success of ipilimumab in 2011 propelled ICI into clinical use and shifted the standard-of-care of advanced melanoma management. The anti-CTLA-4 monoclonal antibody directed against the inhibitory receptor exempts the inhibitory effect of CTLA-4 activation, resulting in activation of T lymphocytes and thus the destruction of tumor cells. Even though ORRs achieved with ipilimumab are below 20%, some patients experience long-term survival using this anti-CTLA-4 regimen. In pretreated CM patients with advanced disease, for example, ipilimumab significantly increased median OS when compared to the peptide vaccine gp100 (10.6 versus 6.4 months). After three years, the OS rate was about 20% followed by a plateau of the survival curve for up to 10 years. Recently, a randomized placebo-controlled study in stage III patients with CM demonstrated that adjuvant ipilimumab increases relapse-free survival (RFS) and OS. Nevertheless, more than half of patients developed severe side effects (grade 3 or 4) with ipilimumab, and five patients (1.1%) even had a grade 5 outcome [23,24,25,26,27]. As a result of ipilimumab-induced T cell activation, a variety of immune-mediated adverse effects (irAEs) have been observed, particularly including colitis, skin rashes, hepatitis, and less frequently hypophysitis.

**Figure 2:**
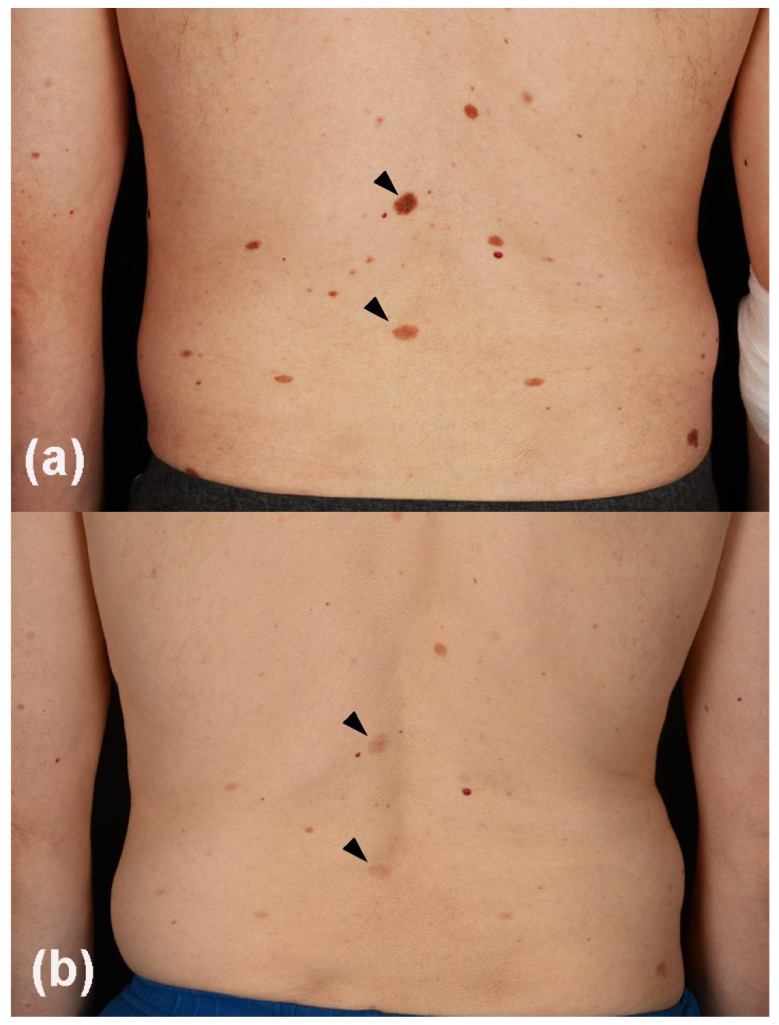
Showing a 59-year old male patient with metastatic melanoma and multiple benign melanocytic nevi on the back (**a**). After 4 cycles (3 months) of anti-PD-1 plus anti-CTLA-4 combination therapy, he developed marked nevi lightening (**b**, highlighted by arrowheads).

After the anti-CTLA-4 proof of concept showing that checkpoint blockade is in fact a beneficial approach to combat CM, pembrolizumab, and nivolumab were studied in this condition. These two monoclonal antibodies against PD-1 were approved by the FDA in 2014, becoming the first-line treatment option in metastatic melanoma. Large, randomized investigations have demonstrated that mono-nivolumab or -pembrolizumab were superior to mono-ipilimumab. Mono-pembrolizumab in the management of naïve as well as pretreated patients resulted in sustained ORR of 30 to 40% [27,28,29]. In previously untreated CM patients, pembrolizumab revealed OS rates of 51% and 41% after three and five years, respectively. Studies of mono-nivolumab demonstrated ORR of 32% in treatment naïve patients and 40% in pretreated CM. The three-year OS rate for mono-nivolumab in therapy-naive patients was 42%, while the five-year OS in pretreated CM patients with this monotherapy was 35%. Cross-trial comparisons of homogeneous groups of CM patients with mono-pembrolizumab or -nivolumab treatment revealed similar results with respect to the clinical endpoints and side effects [27,28,29,30]. Furthermore, adjuvant monotherapy using nivolumab or pembrolizumab is now the preferred treatment option in patients with resected stage III disease, in particular, stage IIIB to IIID. Compared to ipilimumab, nivolumab has improved RFS with lower rates of adverse events. In this clinical setting, pembrolizumab therapy revealed significantly longer RFS than placebo [31]. Moreover, neoadjuvant melanoma trials, also including immunotherapies, currently investigate agents with promising clinical and biomarker results [32]. As previously mentioned, however, ICI can produce a wide range of irAEs affecting a multitude of organs such as skin, gastrointestinal tract, endocrine system, heart, lung, kidneys, and the nervous system. The most frequent irAEs induced by anti-PD-1 agents are hypo- or hyperthyroidism, pneumonitis, cutaneous reactions (Figure 2), including severe conditions (Stevens-Johnson syndrome, etc.), and hepatitis. irAEs may develop at any time as indicated by a wide range of first occurrence for different organs (e.g., from few days after initiation up to 15 months for skin or up to 12 months for the gastrointestinal tract) [33,34]. In general, oral and intravenous corticosteroids are the mainstay of irAEs management. Depending on the irAEs, their severity and non-responsiveness to corticosteroids, other immunomodulatory, and immunosuppressive drugs may be indicated.

**Figure 3:**
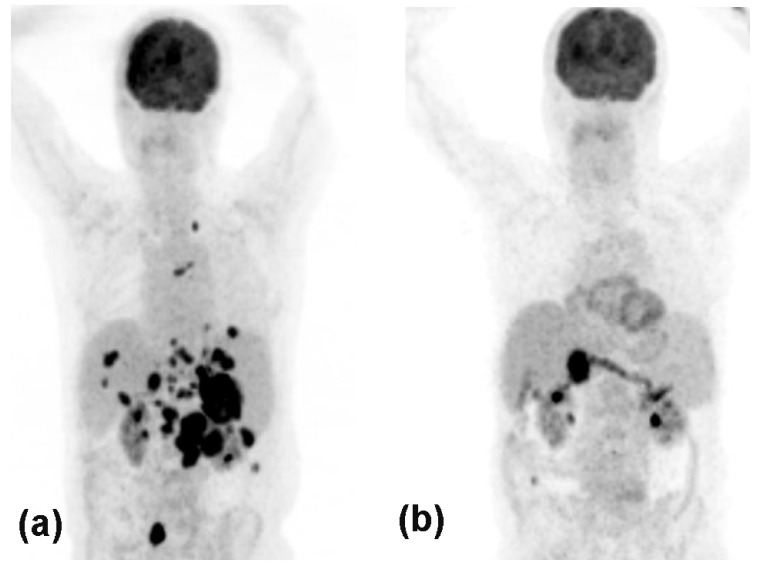
PET-CT of an 80-year old male showing massive melanoma metastases in the entire abdomen (**a**), which almost completely resolved after two cycles of anti-PD-1 plus an-ti-CTLA-4 combination therapy (**b**).

With the attempt to further increase the number of patients who benefit from immunotherapy, combination therapies using anti-CTLA-4 and anti-PD-1 antibodies have been studied in large trials. Two studies demonstrated that the combination of nivolumab plus ipilimumab (ORR: 56.7%) resulted in higher clinical benefit when compared to -nivolumab (ORR: 43.7%) or mono-ipilimumab (ORR: 19%). Five-year OS rates were 52% in the combined treatment arm, 44% in the mono-nivolumab arm, and 26% in the mono-ipilimumab arm [26,27,28,29]. In 2015, the combination of nivolumab plus ipilimumab was approved based on positive ORR and PFS data. In case of primary or secondary resistance to anti-PD-1, monotherapy combination or mono-ipilimumab is a potential therapeutic approach. Today, the respective benefits of combination ICI versus sequential ICI are still unclear. Combination therapies are associated with much higher rates of irAEs, which are justified by long-term disease response. However, the subgroup of patients who might benefit from the combination is not known prior to therapy, potentially exposing patients to unnecessary toxicity [25,26,27,28,29,30,31]. Brain metastases are a common cause of disabling neurologic complications and poor prognosis in CM patients. In a phase 2 trial, patients with small, untreated, and asymptomatic brain metastasis were enrolled—it was demonstrated that ipilimumab plus nivolumab have clinically meaningful intracranial efficacy (56% of intracranial response). The safety profile was similar to those reported for the combination in patients without brain metastasis [35]. Another phase 2 clinical trial compared the combination of nivolumab plus ipilimumab versus nivolumab alone. Despite the small sample size, ICIs combination was superior to nivolumab monotherapy, with a higher proportion of patients achieving intracranial response [35]. An exemplary case shows almost complete remission after ICI combination treatment is depicted in Figure 3. However, the combination of anti-PD-1 and anti-CTLA-4 agents is associated with even higher irAEs and drop-out rates when compared to monotherapy regimens. Hence, ICI can exhibit a variety of irAEs, which range in severity but can have detrimental effects on a patient’s quality of life and well-being, limiting subsequent treatment options [36]. Combinations of two or more therapies (e.g., BRAF/MEKi, T-VEC, or anti-angiogenic modulators) with ICI, although potentially leading to improved efficacy, can increase the incidence and severity of irAEs. Overcoming irAEs thus presents a major challenge in immunotherapeutic and combination approaches to target CM.

Despite recent advances in ICI therapy in CM, a subgroup of patients does not respond to ICI treatment, with a significant portion of patients relapsing within 2 years. ICI resistance can be caused by a variety of factors, including an immunosuppressive tumor microenvironment, levels of tissue-specific neoantigens, altered tumor-infiltrating lymphocyte function, and specific oncogenic alterations in the heterogenous tumor entities [37,38,39]. Therefore, regimens are being studied, including new immunomodulators and combination treatments using ICI with targeted therapy, such atezolizumab, vemurafenib, and cobimetinib. Currently, in-use ICIs and BRAF/MEK inhibitors are summarized in Table 1. Furthermore, evidence is accumulating on the use of new immunomodulatory treatments, for example, addressing LAG3, TIM3, and GITR [16,40].

## 3. Targeted Therapy

Pharmacological approaches to target skin cancer have been widely studied in the last two decades. Targeting cancer by focusing on cancer type-specific genetic alterations and the most consequential signaling cascade abnormalities in tumor initiation and progression are direct and profound applications to rehabilitate cellular homeostasis. In NMSC, several critical pharmacological modulators are known to exhibit favorable effects. Targets of systemic therapies include COX2, Toll-like-receptors (TLR), growth factor receptors such as EGFR and the sonic hedgehog, and m-TOR signaling pathways [41,42,43,44,45,46].

**Figure 4:**
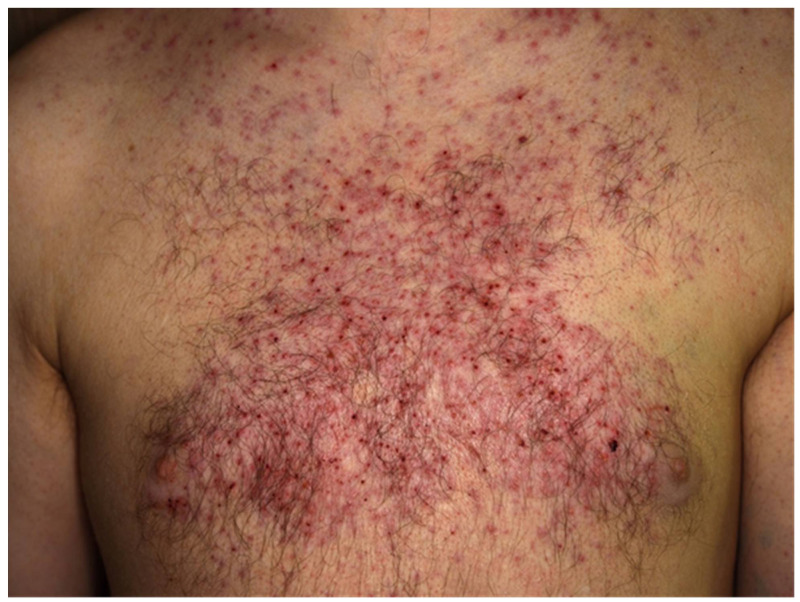
Widespread acneiform rash developing 2 months after the initiation of anti-BRAF plus anti-MEK combination therapy in a 33-year-old male with metastatic melanoma.

In advanced melanoma, the most common druggable mutations are found in the MAPK/ERK pathway [47,48] (Figure 1b). Up to half of the patients diagnosed with CM carry activating mutations in the serine-threonine kinase of the BRAF gene (BRAF-V600), with almost a quarter of patients (15–25%) carrying mutations in the RAS gene (Q61R, Q61K), which downstream activate RAF, MEK, and ERK. [49,50,51]. Other mutations associated with poor outcomes include CDKN2A and TP53 in ~13% and ~15% of melanoma patients, respectively [52]. MAPK/ERK pathway mutations enhance proliferation, survival, and spread of melanoma cells, and thus, patients carrying the mutation are eligible for treatment with BRAF and MEK inhibitors. In BRAF-mutant metastatic melanoma, BRAF and MEK inhibitors have proven to improve survival, although half of the patients develop resistance within a year [53,54]. Moreover, treatment with BRAF and MEK inhibitors is associated with toxicity, as shown in a case of widespread acneiform rash developing 2 months after the initiation of anti-BRAF plus anti-MEK combination therapy (Figure 4). Several adverse events (AE), including hyperkeratosis, rash, alopecia, skin papilloma, palmar-plantar hyperkeratosis, and arthralgia, as well as rare adverse events such as cutaneous SCC and pyrexia were observed. Most used BRAF/MEK inhibitor combinations include Dabrafenib/Trametinib, Vermurafenib/Cobimetinib, and Encorafenib/Binimetinib. In addition, BRAFi was associated with increased antigen expression, lymphocyte homing, and a decrease in immunosuppressive cytokine release in melanoma cell lines and patients’ biopsies, providing a rationale for ICI-BRAF/MEKi combinations [55,56,57,58]. This was further explored in a first-in-human clinical trial of dabrafenib, trametinib, and pembrolizumab in which 11/15 patients (73%) showed an objective response and 6/15 continued with a response at a median follow-up of 27 months. Triple therapy had higher PFS (16 months) compared to dabrafenib and trametinib double therapy (10.3 months) with a median duration of 18.7 months. [58,59]. Recently, a randomized, double-blind phase 3 trial on 514 patients suffering from stage III-IV BRAFV600-positive melanoma evaluated the use of atezolizumab in combination with vermurafenib and cobimetinib. Progression-free survival was significantly prolonged from 10.6 months in the control group to 15.1 months in the atezolizumab group with similar ORR (65% vs. 66%) [60]. While triple therapy combinations were prone to a higher incidence of AEs, these studies were the first to indicate that BRAFi/MEKi/anti-PD1/PDL1 combinations have the potential to increase the frequency of long-lasting antitumor responses in BRAF v600-mutant melanoma patients [58,60].

**Figure 5:**
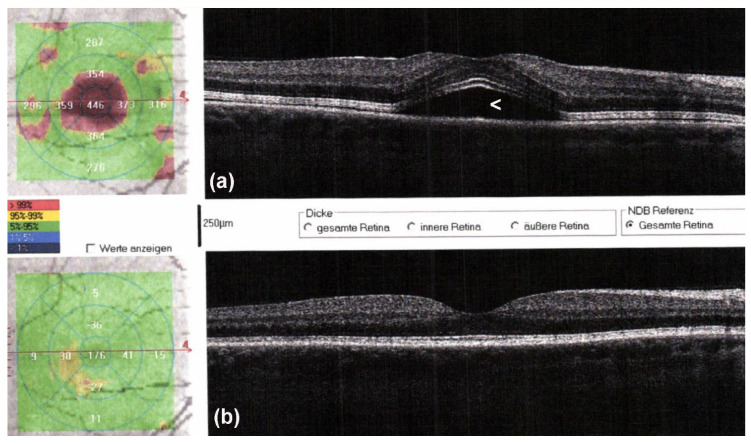
Optical coherence tomography (OCT) scans of a male patient with metastatic melanoma started targeted therapy with encorafenib and binimetinib. Two days after therapy initiation, he noticed blurry vision. On OCT, serous retinal detachment with subfoveolar fluid (**a**, arrowhead) is observed, which spontaneously resolved after withholding targeted therapy for 5 days (**b**).

As with ICI, targeted therapies are also associated with AEs occurring of any grade in almost all patients treated with the combination therapy. In patients treated with BRAFi/MEKi, grade 3 to 4 AEs have been observed in about 50%. The discontinuation rate due to AEs of BRAFi/MEKi is about 15% and thus much lower as compared to combinations of anti-PD-1 and anti-CTLA-4. The most frequently reported AEs of BRAFi/MEKi include cutaneous toxicities (Figure 4; e.g., acneiform rashes, photosensitivity, palmoplantar hyperkeratosis), diarrhea, pyrexia, hepatic toxicities, arthralgia, cardiovascular toxicities (e.g., hypertension, QT-prolongation), ocular AEs (Figure 5; retinal detachment, uveitis), and rarely pneumonitis.

## 4. Intratumoral Tumor Therapy and Combinations

Oncolytic Viral Therapy (OVT) utilizes replication-competent native or genetically engineered herpes and adenoviruses to selectively target, infect, and lyse tumor cells. Moreover, oncolytic viruses have the potential to be used as vehicles for transduction of immunomodulatory transgenes in a tumor-promoter-driven system to enhance anti-tumor immunity and assist in immune checkpoint blockade (Figure 1c). The first oncolytic virus approved in 2015 for melanoma treatment was the engineered herpes-simplex virus 1 (HSV-1) Talimogene laherparepvec (T-VEC). Deletion of RL1 and US12, encoding for ICP34.5 and ICP47 in T-VEC blocks the ability of the virus to hijack the replication machinery of normal cells by making it susceptible to the anti-viral cell response through protein kinase R (PKR) activation. Tumor cells, due to a disrupted PKR and IFN pathway, thus become main targets for infection and lysis by T-VEC. Moreover, it was found that insertion of US11 and GM-CSF improved oncolytic effect and augmented immune response leading to systemic activation of CD8+ TIL [61,62,63,64,65]. Several reports have shown intratumoral injection of T-VEC to be effective in the treatment of recurrent and locally advanced Merkel cell carcinoma (MCC) [66,67]. A recent report of T-VEC and concurrent pembrolizumab treatment led to a complete response in 51-year old patients with refractory MCC [68]. Two cases showed a complete and partial response in two 69-year old and 76-year-old Caucasian males with pre-treated anti-PD-1 refractory MCC after concurrent PD1/PDL1 inhibition and T-VEC treatment [69]. Nevertheless, T-VEC/ICI combinations in MCC treatment remain largely under-studied. In CM, several clinical studies were published showing good efficacy and tolerability, with few reported severe AEs of T-VEC treatment in patients with stage III-IV melanoma [70,71,72,73,74]. Recently, final analysis of the OPTiM phase III trials comparing T-VEC versus GM-CSF treatment in 436 patients with unresectable stage III-IV melanoma, T-VEC was shown to improve long-term efficacy and was well tolerated with median OS for T-VEC treatment of 23.3 months and ORR of 31.5% [72]. Combining T-VEC and ICI or BRAF/MEK inhibition in CM treatment are being widely studied [61,63]. T-VEC can prime the anti-tumor response and turn immunologically ‘cold tumors’ to become ‘hot’ by induction of IFN signaling overcoming immunosuppression of the TME. Moreover, T-VEC can induce PD-1 expression in tumor cells, making them susceptible for ICI and viral GM-CSF, and chemokine release can lead to attraction and maturation of APCs that can cross prime CD8+ TIL in anti-tumor response. First, phase I/II studies on T-VEC/ipilimumab or pembrolizumab combinations show higher effectiveness of T-VEC/ICI combinational therapy versus T-VEC monotherapy in stage I/II melanoma [74]. Patients receiving ipilimumab/T-VEC combinations experienced a higher incidence of pseudo-progression, and combinational therapy was associated with higher ORR (39%) [75]. Similarly, pembrolizumab/T-VEC combinations were well tolerated with an ORR of 62% [61]. Another oncolytic adenovirus, ONCOS-102, that was engineered to express GM-CSF, in combination with pembrolizumab, showed promising results in melanoma mouse models and is currently undergoing a pilot study in advanced melanoma patients after anti-PD1 treatment [76] (NCT03003676). 

In a pre-clinical study, MAPK inhibition was shown to enhance T-VEC replication in murine and human melanoma cell lines. BRAFi leads to enhanced T-VEC oncolysis in BRAF-mutated melanoma lines, while MEKi increased T-VEC effectiveness in both BRAF-mutated and BRAF-wildtype cell lines [63]. Thus, combining T-VEC and BRAF/MEK inhibition might represent a potentially promising avenue to enhance T-VEC efficacy in BRAF-mutated and BRAF-wildtype melanoma and requires further pre-clinical and clinical validation. 

Cytokines such as interleukin 2 (IL-2) and granulocyte-macrophage colony-stimulating factor (GM-CSF) were among the first intratumoral regimens assessed in melanoma [77,78] (Figure 1d). In the USA, systemically administered IL-2 is approved for the treatment of metastatic CM. Even though the anti-tumor efficacy of intratumoral IL-2 appears to be durable, it is limited to the injected lesions suggesting that intratumoral IL-2 does not have strong systemic effects. Unlike systemic IL-2, however, intratumoral IL-2 is generally well tolerated [79,80]. In a phase 2 study, tavokinogene telseplasmid—a synthetic plasmid encoding the cytokine IL-12—demonstrated the induction of an anti-tumor immune response and a high control rate in patients with CM. In 2017, this drug was given an orphan drug status in the USA for the management of unresectable metastatic melanoma [81]. Another type of intratumoral approach in development comprises the pattern recognition receptor (PRR) agonists, including Toll-like receptor (TLR) agonists and stimulator of interferon genes (STING) agonists. Three TLR-9 agonists, such as SD-101, IMO-2125, and CMP-001, are under investigation in combination treatments [82]. Moreover, it has been detected that cyclic dinucleotides may represent immune adjuvants by activating STING, in turn stimulating a pro-inflammatory immune response. Hence, phase 1 studies on 2 intratumoral STING agonists have been initiated [79,83]. A synergy between intratumoral agents and ICI may be expected. In fact, treatment regimens combining therapies that have different modes of action without enhancing toxicity are probable to feature in future investigations [79]. There is increasing data indicating that the combination of intratumoral agents and systemic regimens can even achieve responses in anti-PD1-refractory cancers, thereby overcoming resistance. Importantly, intratumoral strategies may be considered for use in any cancer that is injectable [79].

## 5. Potential of Anti-Angiogenic Immunotherapy in CM Treatment

The role of tumor angiogenesis in cancer has been known for nearly 50 years [84,85]. Under homeostatic conditions, blood vessels are considered highly organized, specialized systems with organ-specific functions [86]. Disruption of pro- and anti-angiogenic balance in tumor angiogenesis can lead to unorganized and permeable vessels lacking proper barrier function. This, in turn, can increase the spread and risk of metastasis formation, which has detrimental effects on patient survival outcomes. Abnormal angiogenesis is at the core of melanoma growth and metastasis formation, with melanoma carcinogenesis being highly dependent on the recruitment of blood vessels from the periphery by promoting angiogenesis through the release of vascular endothelial growth factor (VEGF) [87,88]. 

In an advanced setting, melanoma cells are capable of hijacking angiogenic programs, which can promote metastatic dissemination through lymphatic and hematogenous routes [89,90,91]. Still, anti-angiogenic monotherapy, although improving disease-free interval (DFI), did not prove to have durable and substantial anti-tumor activity in CM [92]. Additionally, VEGF decreases during aging leading to a poorer response in older compared to young patients treated with anti-VEGF inhibitors such as bevacizumab [93]. VEGF is reported to contribute to ICI resistance in mice by reducing CTL trafficking into the TME whilst favoring Treg infiltration through the permeable endothelium [94]. Furthermore, VEGF levels are found to be higher in ICI non-responders than responders indicating an immuno-suppressive function. Moreover, it is reported that high VEGF concentrations induce inhibitory receptor expression forcing CTL exhaustion, decreasing ICI effectiveness [95,96]. Although the immunomodulatory effect of angiogenic molecules such as VEGF and angiopoietins, which can control immune trafficking through regulation of adhesion molecules, was known for the last decade, the emergence of ICI and the challenge of therapeutic resistance in a subset of patients made angiogenic inhibitors potential targets of interest to support ICI and overcome treatment resistance [97,98,99]. Moreover, the dysfunctional tumor vasculature can play a crucial role in immune evasion by cancer cells using an angiogenesis-induced endothelial immune cell barrier hampering antitumor immunity. It was thus suggested that exposure to pro-angiogenic factors leads to endothelial cell anergy, reduced upregulation of endothelial adhesion molecules, and thus decreased leukocyte adhesion, extravasation, and immune infiltration [100,101,102]. Hence, vessel normalization and immune modulation by angiogenic inhibitors exhibit synergistic effects that can potentiate cancer immunity and the anti-tumor response of ICI [103,104,105].

In recent years, the use of ICI in combination with anti-angiogenic modulators such as bevacizumab was widely studied in several solid tumors, including non-small-cell lung cancer [106], renal carcinoma [107,108], hepatocellular carcinoma [109,110], and endometrial cancer [33]. Moreover, recently a landmark phase III randomized clinical study in which patients with unresectable hepatocellular carcinoma were treated with a PD-L1 inhibitor (atezolizumab) and a VEGF inhibitor (bevacizumab) combination found a significantly improved overall and progression-free survival when compared to a group treated with the standard of care protein kinase inhibitor, sorafenib [34]. Combinations of ICI with systemic and local treatments for HCC are already under evaluation in large-scale clinical trials, and atezolizumab/bevacizumab combinations are predicted to soon become the standard of care as first-line therapy in HCC [111]. 

In CM, pre-clinical studies have previously suggested that angiopoietin-2 and VEGFA inhibition by a bispecific antibody can elicit antitumor immunity and enhance PD-1 blockade in a tumor transplant mouse model of melanoma [112]. Remarkably, in BRAF-mutated melanoma, VEGF blockade was even suggested to benefit long-lasting tumor responses, delay onset of BRAFi resistance, and induce macrophage infiltration [113]. Although adjuvant bevacizumab treatment was demonstrated to not significantly affect 5-year disease-free survival in resected melanoma with a high-risk of recurrence, several studies are currently evaluating the safety and efficacy of ICI/bevacizumab combinations [92]. A phase I trial in advanced melanoma patients has shown VEGF-A blockade with ipilimumab administration to be safe with increased CD8 T cell and macrophage infiltration in tumor biopsies as well as improved survival with a median of 25.1 months [113,114]. Two phase II studies evaluating ipilimumab monotherapy vs. ipilimumab/bevacizumab in unresectable stage III/IV melanoma and atezolizumab/bevacizumab in patients with locally advanced and metastatic cutaneous and mucosal melanoma are currently ongoing and recruiting (NCT01950390/NCT04091217). In addition, two phase I trials evaluating pembrolizumab plus angiopoietin-1/-2-neutralizing peptibody, AMG386, as well as tremelimumab plus anti-angiopoietin-2 antibody, MEDI3617, are recruiting and ongoing (NCT03239145/NCT02141542). Overall anti-angiogenic inhibitors have the potential to support ICI immunotherapy and help in overcoming treatment resistance in advanced melanoma patients. A summary of currently studied therapeutic combinations for advanced melanoma treatment is depicted in Table 2.

## 6. Conclusions and Future Perspective

The development of patient-centered approaches for targeted treatment will be one of the main challenges in CM care in the next decade. Combining therapeutic options offers the unique opportunity to tailor treatment to patient- and cancer-specific conditions utilizing the synergistic effects of existing therapeutic approaches (Figure 1e). Combinations of several systemic treatment options such as ICI, BRAFi/MEKi, and angiogenic inhibitors for metastatic melanoma, as well as intratumoral and systemic immunotherapeutic applications to prime and induce immuno-sensitivity of locally advanced CM show promising results and potential to overcome acquired and endogenous treatment resistance. Nevertheless, limitations for increasingly complex treatment combinations remain. Patients treated with ICI can exhibit a range of non-cutaneous and cutaneous irAEs, which range in severity and can have detrimental effects on a patient’s quality of life and well-being, limiting subsequent treatment options [36]. Combining two or more therapeutic approaches, although potentially leading to improved efficacy, can increase the incidence and severity of irAEs. As discussed in previous sections, there is a range of potentially life-threatening irAEs affecting a multitude of organs, but there are also less severe cutaneous irAEs substantially impacting patient’s quality of life. Cutaneous irAEs are the most common adverse events occurring in up to 50% of patients undergoing ICI resemble autoimmune disorders mostly presenting as primary dermatoses [115,116,117]. Future investigations will have to address emerging non-cutaneous and cutaneous AEs as well as potential augmentation of treatment interventions combining different modes of action whilst minimizing toxicity. In addition, we would like to emphasize that the survival of patients with CM is significantly improved when the tumor is detected at a very early stage—a time when simple surgery is fully sufficient to cure the patient’s CM. Hence, we also want to refer to emerging novel techniques under current investigation, which might significantly improve the accuracy of early CM diagnosis. A variety of innovative optical and acoustic technology-based techniques, such as confocal laser-scanning microscopy, optical coherence tomography (OCT), photoacoustic/ultrasound/OCT, multiphoton excited fluorescence imaging, and stepwise two-photon excited fluorescence, have been developed to increase the diagnostic accuracy for the non-invasive melanoma diagnosis [118,119,120,121,122,123,124]. Advancing diagnostic tools and biomarkers to identify subgroup beneficiaries of specific treatment combinations prior to therapy can thus become the next step in patient-centered personalized CM treatment.

## Figures and Tables

**Table 1:** Currently in-use ICIs and BRAF/MEK inhibitors for CM.

Biological Classification	Drug Name	Mechanism	Main Target	Study
Immune Checkpoint Inhibition (ICI)				
Monoclonal Antibody	Ipilimumab	priming of anti-tumor immune response by checkpoint receptor inhibiton on immune and melanoma cells	CTLA4	NCT00094653
	Pembrolizumab		PD1	KEYNOTE-001 KEYNOTE-002 KEYNOTE-006
	Nivolumab		PD1	NCT01844505
	Atezolizumab		PDL1	IMspire150 NCT02908672
Targeted Therapy				
Kinase Inhibitor	Dabrafenib	inhibition of MAPK/ERK overactivation in BRAF-mutant melanoma	BRAF mutant	BREAK-2 BREAK-3
	Vermurafenib			NCT01689519
	Encorafenib			NCT01909453
	Trametinib		MEK	METRIC MEK113583
	Cobimetinib			NCT01689519
	Binimetinib			NCT01909453

**Table 2:** Combinational treatment strategies for CM.

Biological Classification	Drug Name	Mechanism	Main Target	Study
**Combined Targeted Therapy**				
**Kinase Inhibitor**	Dabrafenib + Trametinib	inhibition of BRAF-MEK pathway reactivation, decreased tumor cell survival in BRAF-mutants	BRAF mutant, MEK	NCT01584648 NCT0159790
	Vermurafenib + Cobimetinib			NCT01689519
	Encorafenib + Binimetinib			COMBO450
ICI + Combined Targeted Therapy				
Monoclonal Antibody + Kinase Inhibitor	Pembrolizumab + Dabrafenib + Trametinib	PD1 checkpoint blockade, decreased tumor cell survival in BRAF-mutants	PD1, RAF, MEK	NCT02130466
	Atezolizumab + Vermurafenib + Cobimetinib	PDL1 checkpoint blockade, decreased tumor cell survival in BRAF-mutants	PDL1, RAF, MEK	NCT02908672 IMspire150
Intratumoral Stimulation				
Cytokine	GM-CSF	stimulation of tumor immune response by GM-CSF	APCs, TILs	E4697
	IL-2	stimulation of tumor immune response by IL2	TILs, NK cells	NCT01672450 NCT00204581
Oncolytic Virus	Talimogene laherparepvec (T-VEC)	oncolysis, activation of anti-tumor immune response through IFN signalling	tumor cells, innate and adaptive Immunity	NCT00769704
	ONCOS-102	oncolysis, immune activation by GM-CSF	tumor cells, innate and adaptive Immunity	NCT03003676
Plasmid	Tavokinogene telseplasmid	stimulating diffentiation, activation of the adaptive immune system	APCs, TILs	NCT01502293
TLR9 agonists	SD-101; IMO-2125; CMP-001; CPG 7909	induction of CD8 T cell response enhancing uptake, destruction of cancer cells	TLR9 in APCs, TILs	NCT02521870 NCT02644967 NCT02680184
STING agonists	ADU-S100, MK-1454	anti-tumor response through type I IFN signalling activation	STING in tumor cells, TIL, APCs	NCT02675439 NCT03172936 NCT03010176
Immune Checkpoint Inhibition + Intratumoral Stimulation				
Oncolytic Virus + Monoclonal Antibody	T-VEC + Ipilimumab	immunological priming of TME to enhance PD-1 blockade efficacy	tumor cells, TILs, APCs	NCT01740297
	T-VEC + Pembrolizumab			MASTERKEY265 KEYNOTE034
	ONCOS-102 + Pembrolizumab			NCT03003676
Intratumoral Stimulation + Targeted Therapy				
Oncolytic Virus + Kinase Inhibitor	T-VEC + Trametinib	oncolysis, adaptive immune activation, MEK inhibition to reduce tumor cell survival	tumor cells, MEK, TILs and APCs	NCT03088176
ICI + Anti-Angiogenesis				
Monoclonal Antibody	Bevacizumab	vessel normalization, immunomodulation, checkpoint inhibition	VEGF	AVAST-M
	Ipilimumab + Bevacizumab		CTLA4, VEGF	NCT01950390
	Atezolizumab + Bevacizumab		PDL1, VEGF	NCT04091217
Monoclonal Antibody + Neutralizing Peptibody	Pembrolizumab + AMG386	vessel normalization, immunomodulation, checkpoint inhibition	PD1, Angpt1/2	NCT03239145
	Tremelimumab + MEDI3617		CTLA4, Angpt2	NCT02141542

## Data Availability

All data generated or analyzed during this study are included in the published article.

## Abbreviations

AE: Adverse events; irAE: Immune-related adverse events; Blimp-1: B lymphocyte-induced maturation protein 1; BRAFi: BRAF inhibition; CM: Cutaneous melanoma; CTLA4: Cytotoxic T-Lymphocyte Associated Protein 4; CTL: Cytotoxic T-Lymphocyte; GM-CSF: Granulocyte-macrophage colony-stimulating factor; ICI: Immune checkpoint blockade; ITIM: Immunoreceptor tyrosine-based inhibitory motif; KLRG-1: Killer cell lectin-like receptor sub family G; LAG3: Lymphocyte-activation protein 3; MAPK: Mitogen-activated protein kinase; MCC: Merkel cell carcinoma; MEKi: MEK inhibiton; NSCLC: Non-small-cell lung cancer; OVT: Oncolytic viral therapy; OS: Overall survival; ORR: Overall response rate; PD-L1: Programmed cell death ligand 1; PFS: Progression-free-survival; PKA: Protein kinase A; PRR: Pattern recognition receptor; RFS: Relapse-free survival; T-VEC: Talimogene laherparepvec; TCR: T-cell receptor; TIGIT: T cell immunoreceptor with Ig and tyrosine-based inhibitory motif (ITIM) domains; TIM3: T cell immunoglobulin domain and mucin domain-containing protein3; TLR: Toll-like receptor; TME: Tumor microenvironment; Treg: Regulatory T; UV: Ultraviolet; VEGF: Vascular endothelial growth factor.
